# The Impact of Gamified Teaching on Undergraduate Nursing Students’ Disaster Nursing Competence, Self-Efficacy, and Self-Directed Learning Ability: Quasi-Experimental Study

**DOI:** 10.2196/85947

**Published:** 2026-06-10

**Authors:** Shiyi Bai, HuiJuan Zeng, Qianmei Zhong, Cheng Yang, Shenglu Liu, Lulu Cao, Mei He

**Affiliations:** 1 Mianyang Central Hospital, School of Medicine, University of Electronic Science and Technology of China, No 12 Changjiaxiang, Fucheng District, Mianyang City, 621000, China, 86 13778440262; 2 Sichuan Institute of Industrial Technology, Deyang, China

**Keywords:** gamified teaching, nursing education, undergraduate nursing students, quasi-experimental study, disaster nursing

## Abstract

**Background:**

Gamified teaching is considered an effective strategy for enhancing learning motivation. However, there is insufficient research on systematically designing theory-based gamified approaches for disaster nursing, particularly in resource-limited conventional classroom settings.

**Objective:**

This study aimed to compare the impact of gamified teaching versus conventional teaching on disaster nursing competence, self-efficacy, and self-directed learning ability among undergraduate nursing students.

**Methods:**

A quasi-experimental, nonequivalent control group pretest-posttest design was used. Participants were third-year nursing students who had completed foundational and medical-surgical nursing courses with no prior gamified learning (GL) experience. They were nonrandomly allocated to experimental (GL group, n=66) and conventional learning (CL group, n=66) groups based on scheduling feasibility. The GL group received 180 minutes (4 class hours) of gamification, incorporating points, group competitions, and collaboration. The CL group received traditional instruction. Outcomes were measured at 3 time points (preintervention, immediate posttest, and 1-week follow-up) using the Disaster Nursing Competence Scale, General Self-Efficacy Scale, and Self-Directed Learning Ability Scale of Nursing Undergraduates. Data were analyzed using linear mixed models with group, time, group × time interaction, and baseline differences (disaster experience) as covariates.

**Results:**

The GL group scored significantly higher than the CL group on the total posttest score for disaster nursing competence (mean difference [MD]=0.205, 95% CI 0.032‐0.378; *P*=.02), although the difference was not statistically significant at follow-up. The knowledge dimension (MD=0.274; *P*=.01) and practical skills dimension (MD=0.201; *P*=.03) showed the same pattern of immediate improvement, whereas the physical and mental qualities dimension demonstrated a delayed effect at follow-up (MD=0.537; *P*=.003). The total score for self-directed learning ability was higher in the gamified teaching group than in the control group at follow-up (MD=0.116, 95% CI 0.012‐0.220; *P*=.03), with significant improvements in collaborative learning ability (group × time interaction effect *F*_2,390_=5.17, *P*=.01; follow-up between-group difference=0.243, *P*=.001) and information competence (follow-up between-group difference=0.140; *P*=.04), but no significant change in self-management ability. There were no significant differences between the 2 groups in general self-efficacy at any time point (interaction effect *P*=.33).

**Conclusions:**

Unlike most studies focusing on digital gaming platforms, this study shows that a low-technology, highly interactive classroom gamification approach is feasible under conventional teaching conditions. The uniqueness of this gamified teaching lies in its ability to simultaneously improve multidimensional competencies (knowledge, skills, psychological qualities, self-directed learning, and learning collaboration ability) and reveal a delayed effect on physical and mental qualities—a pattern rarely reported before. This suggests potential utility in resource-limited nursing education and provides a practical, resource-sensitive solution for disaster nursing training. However, the absence of significant effects on general self-efficacy indicates that gamified teaching may not influence all psychological constructs equally. Furthermore, the 1-week follow-up may be insufficient to capture longer-term changes in psychological outcomes, and caution is warranted when generalizing these findings to other educational contexts without adaptation.

## Introduction

The frequency, intensity, and complexity of both natural and man-made disasters continue to escalate, presenting significant threats to global public health systems [1]. According to the definition provided by the United Nations Office for Disaster Risk Reduction (UNISDR), a disaster is defined as a serious disruption of the functioning of a community or a society at any scale due to hazardous events interacting with conditions of exposure, vulnerability, and capacity, leading to one or more of the following: human, material, economic, and environmental losses and impacts [2]. Nurses represent the most critical frontline personnel in disaster response, undertaking essential responsibilities such as providing immediate medical assistance, allocating resources, offering psychological support, and coordinating among multiple teams. They play a vital role in minimizing casualties, alleviating psychological trauma among disaster victims, and enhancing the overall effectiveness of disaster management efforts [3]. However, due to limited knowledge and skills related to disaster response, some nurses may lack confidence in their ability to teach and guide others and may even refrain from participating [4]. Although existing studies generally emphasize the necessity for all nurses to receive systematic training in disaster nursing, significant gaps remain in the actual implementation of disaster nursing education.

As future health care providers, nursing students should be fully prepared during their studies to take on this important responsibility and enhance their disaster literacy so that they can directly participate in frontline rescue missions after entering the workforce [5]. Studies have shown that most nursing students lack a sufficient understanding of the core concepts of disaster nursing. Although they generally have a positive attitude toward disaster preparedness, they lack practical experience and have limited relevant knowledge reserves [6]. Therefore, systematically incorporating disaster nursing into nursing curricula, particularly focusing on cultivating students’ disaster response capabilities, is a key strategy to alleviate manpower shortages during disasters and improve overall emergency response capacity.

Disaster sites typically feature high risk, intense pressure, tight time constraints, and limited resources. Traditional disaster nursing teaching methods rely on theoretical instruction and dynamic case analysis, making it difficult to replicate such complex and dynamic situations [7]. Nursing students passively receive knowledge in the classroom and lack opportunities to practice decision-making in simulated chaotic environments, resulting in a disconnect between theoretical knowledge and practical application when facing sudden events, with generally low self-efficacy [8]. Moreover, due to the incidental nature of disaster events, students find it hard to gain direct experience through clinical internships, placing higher demands on their independent learning ability. Traditional teaching methods are also less effective in stimulating students’ sustained learning motivation. Therefore, how to improve students’ disaster nursing competence within limited class hours while cultivating their self-efficacy and willingness for independent learning has become an urgent issue for nursing educators.

In recent years, with the development of educational technology, gamified teaching has gradually entered the field of medical education as an innovative strategy and a promising teaching method [9]. Gamified teaching does not simply mean playing video games; rather, it introduces game elements (such as time pressure, competition and cooperation, and points rewards) to encourage participants to perform certain actions with a playful mindset. This approach can effectively drive active participation from learners. In the fields of medical and disaster education, gamification has been proven to enhance knowledge, skills, satisfaction, and clinical reasoning ability, as well as strengthen learners’ cooperation awareness, decision-making capabilities, and disaster response skills [10]. Compared with traditional teaching, gamified instruction can boost learners’ intrinsic motivation and engagement through clearly defined progression goals and instant rewards while providing opportunities for active learning through designed game elements [11]. In literature reviews, some studies have systematically summarized the effectiveness of different game intervention methods in disaster education [12]. Their results found that using tabletop games in mass casualty incident triage management can improve triage and treatment speed [13]. Serious games based on disasters and crises are more effective than case-based methods in improving nursing students’ knowledge and skills in crisis management [14]. When situational simulation games are combined with team drills and ethical dilemmas, they can effectively improve the emergency preparedness of first responders [15].

Although the value of gamified teaching in stimulating learning interest has been preliminarily confirmed [16], most existing studies are developed based on digital game models. Due to limitations in time, cost, and resources, this has, to some extent, restricted opportunities for repeated practice and standardized assessment [17]. At present, empirical research exploring the combined impact of gamified teaching on disaster nursing capability, general self-efficacy, and self-directed learning ability in the specific group of nursing students remains relatively scarce. Therefore, there is an urgent need for an accessible and scalable educational approach to supplement traditional teaching.

Therefore, this study, based on the International Council of Nurses (ICN) disaster nursing core competency framework, gamified learning (GL) theory, and constructivist theory, aimed to develop a gamified teaching plan for a disaster nursing course suitable for undergraduate nursing students, serving as a supplementary tool to traditional teaching [18-20]. A quasi-experimental design was used to compare the educational impact of GL with conventional learning (CL) among undergraduate nursing students. Specifically, differences in disaster nursing competency, self-efficacy, and self-directed learning ability between the 2 groups were assessed before the intervention, after the intervention, and 1 week postintervention. The evidence obtained from this research can provide practical reference for integrating gamified teaching and serve as a basis for optimizing the design of disaster nursing courses and promoting innovation in nursing education.

## Methods

### Study Design and Setting

This study used a nonrandomized, 2-group, pretest-posttest quasi-experimental design. Due to constraints in teaching arrangements, 2 intact classes were assigned to the intervention group and the control group, respectively, without individual randomization. The intervention group implemented GL, while the control group continued with CL. Both groups were assessed at 3 time points: pretest, posttest, and a 1-week follow-up after the intervention. Quasi-experimental designs do not require individual random assignment and are commonly used in educational research when random scheduling of courses is not feasible. This report follows the Transparent Reporting of Evaluations with Nonrandomized Designs (TREND) statement [21] to enhance transparency and replicability of the research process.

### Participants

#### Sampling Criteria

This study recruited participants using a convenience sampling method. The selection criteria were as follows: (1) third-year students who had completed the courses in fundamentals of nursing, medical-surgical nursing, and the on-campus simulation training assessment (cardiopulmonary resuscitation); (2) no prior experience in GL; and (3) voluntary participation in this study. The recruited nursing students were divided into 2 parallel classes, which were randomly assigned either to the intervention group or the control group, with each group assigned 1 course.

#### Sample Size Calculation

The sample size was calculated using G*Power software (version 3.1.3) [22]. Based on the preliminary results of a pilot study, the parameters were set as 2-tailed *α*=.05, statistical power of 80%, and a medium effect size (Cohen *d*=0.5). Accordingly, the minimum required sample size was 128 participants. A total of 132 undergraduate nursing students were ultimately included.

### Background Setting

The study was conducted at a nursing college in Sichuan Province, China. The Bachelor of Nursing Science program at the college has a 4-year academic duration and includes both the intervention group and the control group students. The study was carried out during the spring semester of 2025, from February 28, 2025, to June 30, 2025.

### Outcomes and Measurement Tools

#### Demographic Variables for Analysis

In general, learners’ age, gender, place of origin, only-child status, prior disaster experience, participation in disaster relief–related training, engagement in on-site disaster first aid, necessity of studying disaster first aid, and familiarity with gamified teaching are used as demographic variables for analysis.

#### Disaster Nursing Competency Scale

The Disaster Nursing Competence Scale was developed by Chinese scholar Yang Meifang [23]. The scale demonstrates high reliability and validity (Cronbach *α*=.97; content validity index=0.94) and has been widely applied among nursing students in China. It contains a total of 47 items covering 3 dimensions: knowledge system, practical skills, and physical and mental qualities. Responses are rated using a 5-point Likert scale ranging from 1 (strongly disagree) to 5 (strongly agree), with higher total scores indicating greater disaster nursing competence.

#### General Self-Efficacy Scale

The General Self-Efficacy Scale (GSES), developed by Schwarzer et al [24], was used. It consists of 10 items rated on a 4-point Likert scale, ranging from 1 (strongly disagree) to 4 (strongly agree). Higher total scores indicate higher levels of self-efficacy. In this study, the Cronbach *α* coefficient was 0.81.

#### Self-Directed Learning Ability Scale of Nursing Undergraduates

This study adopted the “Self-Directed Learning Ability Scale for Nursing Undergraduates” developed by Lin et al [25]. The scale comprises 3 dimensions: self-management ability, information literacy, and cooperation ability, with a total of 28 items. It adopts a 5-point Likert scale (1=strongly disagree to 5=strongly agree), with higher scores indicating stronger self-directed learning ability.

### Disaster Nursing Course

The primary objective of undergraduate nursing students learning the disaster nursing course is to enhance their disaster nursing capabilities. During the first 7 weeks (14 class hours in total, each lasting 45 min), both the experimental group and the control group received unified instruction focused on fundamental knowledge and skills, such as an overview of disaster nursing, medical treatment for various types of disasters, emergency response systems, ethical and legal issues, and occupational protection. During weeks 8 and 9 (4 class hours in total), traditional teaching (CL group) and GL (experimental group) were used for comparative instruction. The course was taught by 2 instructors with extensive experience in disaster rescue and nursing education. To ensure consistency in course delivery, all instructors underwent unified training before the start of the study.

### Intervention

#### Intervention Group

The experimental group received gamified teaching during weeks 8 to 9 (4 class hours). The gamified teaching plan was guided by constructivist theory and GL theory, with teaching content set according to the ICN Disaster Nursing Core Competencies framework [18-20]. The plan clearly defined 3 teaching objectives: knowledge, skills, and emotional attitudes. The total duration of the intervention was 180 minutes, divided into 4 stages according to the teaching logic: disaster preparedness training camp (60 min), on-site disaster practice (40 min), recovery and reflection (40 min), and prevention and community action (40 min). For teaching organization, students were divided into 8 groups using a competitive, task-driven learning model. Every 2 groups formed a competition unit, jointly undertaking the core tasks of a certain stage and conducting display confrontations. Other groups participated in interactive evaluation through observation, questioning, and supplementing to earn points. At the end of the course, the winning group was determined based on the total score. Specific teaching objectives and steps are provided in Multimedia Appendix 1.

#### Control Group

The total duration and stage division of the control group’s course were the same as those of the experimental group (uniform basic teaching for the first 7 wk and 4 class hours during weeks 8 and 9). However, during weeks 8 to 9, the control group received traditional teaching without any gamification elements (such as points, leaderboards, group competitions, or role-playing activities). CL was conducted through teacher lectures, static case analyses, and after-class exercise discussions, with each class lasting 45 minutes. Students continued attending classes in their original groups, without additional group competitions or scenario simulation activities.

### Data Analysis

Data analysis was conducted using IBM SPSS software (version 26.0) [26]. Descriptive statistics were used to summarize demographic and outcome indicator data. Chi-square tests (Fisher exact test used when expected frequencies were <5) were applied to compare baseline differences between the GL group and the CL group. To assess the effects of the intervention on disaster nursing competence, self-efficacy, and self-directed learning ability, linear mixed models were fitted [27]. Each model included a random intercept for student identification to control for correlations arising from repeated measurements at 3 time points (pretest, posttest, and 1-week follow-up). Fixed effects included group, time, group × time interaction, and “disaster experience” as a covariate because of significant baseline differences between groups. Residuals were modeled using an unstructured covariance structure. Sensitivity analyses using a variance components structure yielded almost identical fixed-effect estimates, supporting the robustness of the results. Pairwise comparisons between groups at each time point were based on estimated marginal means and adjusted using the Bonferroni correction. Results were reported as mean differences (MD), 95% CI, and *P* values. The final dataset contained no missing data. Two-tailed *P*<.05 were considered statistically significant.

### Ethical Considerations

This study was reviewed and approved by the Biomedical Ethics Committee of Mianyang Central Hospital (IRB S20250331-01). All participants signed an informed consent form and were informed that they had the right to withdraw from the study at any time without affecting their academic evaluation. All collected data were anonymized, accessible only to the research team, and stored on encrypted devices. Participants did not receive any form of financial compensation. The manuscript does not contain any images or materials that could identify individual participants.

## Results

### Demographic Characteristics

A total of 132 undergraduate nursing students were enrolled in this study, including 66 in the GL group and 66 in the CL group. The participants were predominantly female (n=106, accounting for 80.3%). There were no significant differences between the 2 groups in terms of age, gender, place of origin, only-child status, disaster training, on-site rescue experience, learning willingness, or understanding of gamification (*P*>.05). Only “experience of a disaster ” showed a significant difference between the 2 groups (GL group: 40/66, 60.6% vs CL group: 52/66, 78.8%; *χ*²_1_=5.165; *P*=.02), so it was controlled as a covariate in subsequent analyses. Detailed data analysis is presented in Table 1.

**Table 1.** Demographic comparison of study participants by groups.

Variables	Total (N=132), n (%)	GL^a^ group (n=66), n (%)	CL^b^ group (n=66), n (%)	Chi-square (df)	*P* value
Age (y)				0.00 (1)	>.99
18‐20	18 (13.6)	9 (13.6)	9 (13.6)		
21‐23	114 (86.4)	57 (86.4)	57 (86.4)		
Gender				0.192 (1)	.66
Male	26 (19.7)	14 (21.2)	12 (18.2)		
Female	106 (80.3)	52 (78.8)	54 (81.8)		
Hometown				1.291 (1)	.26
Rural	92 (69.7)	49 (74.2)	43 (65.2)		
Urban	40 (30.3)	17 (25.8)	23 (34.8)		
Only child				0.039 (1)	.84
Yes	35 (26.5)	17 (25.8)	18 (27.3)		
No	97 (73.5)	49 (74.2)	48 (72.7)		
Disaster experience				5.165 (1)	.02
Yes	92 (69.7)	40 (60.6)	52 (78.8)		
No	40 (30.3)	26 (39.4)	14 (21.2)		
Disaster training				—^c^	.12
Yes	7 (5.3)	1 (1.5)	6 (9.1)		
No	125 (94.7)	65 (98.5)	60 (90.9)		
Field rescue experience				—^c^	>.99
Yes	3 (2.3)	1 (1.5)	2 (3.0)		
No	129 (97.7)	65 (98.5)	64 (97.0)		
Willingness to learn				—^c^	>.99
Yes	129 (97.7)	64 (97.0)	65 (98.5)		
No	3 (2.3)	2 (3.0)	1 (1.5)		
Knowledge of gamification				0.893 (1)	.35
Yes	11 (8.3)	4 (6.1)	7 (10.6)		
No	121 (91.7)	62 (93.9)	59 (89.4)		

a GL: gamified learning.

b CL: conventional learning.

c Fisher exact test was used due to expected frequencies <5; thus, no chi-square value is reported.

### The Impact of Gamified Teaching on Various Abilities

A linear mixed model with student ID as a random intercept was used to analyze changes in the 2 groups of students at different time points, with “whether experienced a disaster” included as a covariate in the model. The results are presented in Tables2  3.

**Table 2.** Fixed effects from linear mixed models for all outcomes^a^.

Outcome	Group, *F* test (*df*), *P* value	Time, *F* test (*df*), *P* value	Group × time, *F* test (*df*)*, P* value	Covariate, *F* test (*df*), *P* value
Disaster nursing competence	1.17 (1, 389), .28	55.76 (2, 389), <.001^b^	3.06 (2, 389), .048^b^	0.08 (1, 389), .78
Knowledge	0.84 (1, 396), .36	65.10 (2, 396), <.001^b^	4.28 (2, 396), .01^b^	0.25 (1, 396), .62
Skills	0.91 (1, 396), .34	51.57 (2, 396), <.001^b^	3.60 (2, 396), .03^b^	0.50 (1, 396), .48
Psychophysical quality	0.67 (1, 396), .41	0.71 (2, 396), .49	5.23 (2, 396), .01^b^	0.38 (1, 396), .54
Self-efficacy	0.30 (1, 132), .59	1.53 (2, 264), .22	1.12 (2, 264), .33	0.27 (1, 132), .60
Self-directed learning ability	3.85 (1, 396), .05	4.28 (2, 396), .02^b^	0.95 (2, 396), .39	0.33 (1, 396), .57
Self-management	0.30 (1, 389), .59	1.30 (2, 389), .27	0.72 (2, 389), .49	1.14 (1, 389), .29
Information literacy	5.88 (1, 396), .02	5.77 (2, 396), .003^b^	0.50 (2, 396), .61	0.01 (1, 396), .94
Learning collaboration	2.04 (1, 390), .13	2.85 (2, 390), .06	5.17 (2, 390), .01^b^	0.17 (1, 390), .68

a All models included a random intercept for student identification and the covariate “disaster experience.”

b *P*<.05.

**Table 3.** Between-group comparisons (GL^a^-CL^b^ group) at each time point^c^.

Outcome and time	MD^d^ (SE; 95% CI)	*P* value
Disaster nursing competence		
Pretest	−0.100 (0.088; −0.273 to 0.073)	.26
Posttest	0.205^e^ (0.088; 0.032 to 0.378)	.02^e^
Follow-up	0.062 (0.088; −0.111 to 0.235)	.48
Knowledge		
Pretest	−0.012 (0.097; −0.202 to 0.178)	.90
Posttest	0.274^e^ (0.097; 0.084 to 0.464)	.01^e^
Follow-up	−0.107 (0.097; −0.297 to 0.083)	.27
Skills		
Pretest	−0.142 (0.093; −0.325 to 0.042)	.13
Posttest	0.201^e^ (0.093; 0.017 to 0.384)	.03^e^
Follow-up	0.098 (0.093; −0.086 to 0.281)	.30
Psycho-physical quality		
Pretest	−0.242 (0.178; −0.591 to 0.108)	.17
Posttest	−0.039 (0.178; −0.388 to 0.311)	.83
Follow-up	0.537^e^ (0.178; 0.187 to 0.886)	.003^e^
Self-efficacy		
Pretest	0.101 (0.083; −0.062 to 0.263)	.23
Posttest	0.048 (0.083; −0.115 to 0.210)	.57
Follow-up	−0.066 (0.083; −0.229 to 0.097)	.43
Self-directed learning ability		
Pretest	0.050 (0.053; −0.054 to 0.154)	.34
Posttest	0.015 (0.053; −0.088 to 0.119)	.77
Follow-up	0.116^e^ (0.053; 0.012 to 0.220)	.03^e^
Self-management		
Pretest	0.081 (0.064; −0.045 to 0.208)	.21
Posttest	−0.022 (0.064; −0.148 to 0.105)	.74
Follow-up	0.001 (0.064; −0.125 to 0.128)	.99
Information literacy		
Pretest	0.101 (0.067; −0.031 to 0.234)	.13
Posttest	0.046 (0.067; −0.087 to 0.179)	.49
Follow-up	0.140^e^ (0.067; 0.007 to 0.272)	.04^e^
Learning collaboration		
Pretest	−0.075 (0.073; −0.219 to 0.069)	.31
Posttest	0.020 (0.073; −0.124 to 0.164)	.78
Follow-up	0.243 (0.073; 0.099 to 0.387)	.001^e^

a GL: gamified learning.

b CL: conventional learning.

c Pairwise comparisons were adjusted using the Bonferroni correction.

d MD: mean differences.

e *P*<.05.

### Disaster Nursing Competency

The interaction effect between group and time for disaster nursing ability scores was significant (*F*_2,389_=3.06; *P*=.048), the main effect of time was significant (*F*_2,389_=55.76; *P*<.001), and the main effect of group was not significant. Further intergroup comparisons showed that the GL scored higher than the CL at posttest (MD=0.205, 95% CI 0.032-0.378; *P*=.02). No statistically significant differences were found at the pretest or at the 1-week follow-up. In the knowledge system dimension, the group × time interaction effect was significant (*F*_2,396_=4.28; *P*=.01), as was the main effect of time. The GL group scored higher than the CL group at posttest (MD=0.274, 95% CI 0.084-0.464; *P*=.01), while differences at pretest and the 1-week follow-up were not significant. In the practical skills dimension, the group × time interaction effect was significant (*F*_2,396_=3.60; *P*=.03), and the main effect of time was significant. The GL group scored higher than the CL group at posttest (MD=0.201, 95% CI 0.017-0.384; *P*=.03), with no statistically significant differences at other time points. In the physical and mental fitness dimension, the group × time interaction effect was significant (*F*_2,396_=5.23; *P*=.01), but neither the main effect of group nor time was significant. At the 1-week follow-up, the GL group scored higher than the CL group (MD=0.537, 95% CI 0.187-0.886; *P*=.003). Differences at pretest and posttest were not significant.

### General Self-Efficacy

For general self-efficacy, the main effects of group and time, as well as the group × time interaction, were not statistically significant (interaction effect: *P*=.33). Comparisons between groups at each time point also showed no significant differences (all *P*>.05).

### Self-Directed Learning Ability for Nursing Undergraduates

For the average scores of autonomous learning ability among nursing undergraduates, the main effect of time was significant (*F*_2,396_=4.28; *P*=.02), and the main effect of group was marginally significant (*P*=.05), whereas the group × time interaction effect was not significant. At the 1-week follow-up, the GL group scored higher than the CL group (MD=0.116, 95% CI 0.012-0.220; *P*=.03). Regarding the self-management ability dimension, the main effects of group and time, as well as the interaction effect, were not significant, and there were no significant differences between groups at any time point. In the information capability dimension, both the main effect of group (*F*_1,396_=5.88; *P*=.02) and the main effect of time (*F*_2,396_=5.77; *P*=.003) were significant, whereas the interaction effect was not significant. At the 1-week follow-up, the GL group scored higher than the CL group (MD=0.140, 95% CI 0.007-0.272; *P*=.04). In the learning cooperation ability dimension, the group × time interaction effect was significant (*F*_2,390_=5.17; *P*=.01), and the main effect of time was close to significance (*P*=.06). At the 1-week follow-up, the GL group scored higher than the CL group (MD=0.243, 95% CI 0.099-0.387; *P*=.001), while there were no significant differences between groups at pretest and posttest.

## Discussion

### Principal Findings

This study adopted a quasi-experimental design to explore the impact of gamified teaching on disaster nursing competence, self-efficacy, and self-directed learning ability among undergraduate nursing students. The results showed that gamified teaching enhanced students’ disaster nursing competence, with the knowledge system and practical skills dimensions exhibiting significant advantages immediately after the intervention (posttest), while the physical and mental quality dimension showed significant improvement only 1 week after the intervention (follow-up). Gamified teaching had no significant impact on general self-efficacy. In addition, gamified teaching improved self-directed learning ability, especially information literacy and collaborative learning ability, which were significantly better than those of the traditional teaching group at follow-up, while self-management ability was unaffected. These findings suggest that gamified teaching can serve as an effective supplementary method for disaster nursing education, but its effects are dimension-specific and time-dependent [16].

### The Impact of a Gamification Teaching Program on Disaster Nursing Ability

This study found that the disaster nursing competency scores of the experimental group were higher than those of the control group, which is consistent with previous studies, indicating that gamified teaching can be used as a feasible and effective disaster nursing teaching method [28-32]. It should be noted that this study integrated game elements into existing teaching activities, rather than developing them into a complete game system. This design aimed to use game elements to enhance learning motivation and engagement, while maintaining the fundamental structure of teaching, consistent with the framework proposed by Deterding [33,34].

However, although the impact of gamified teaching improved at posttest, it gradually faded after 1 week. One possible explanation for this finding is that gamification, as a nontraditional teaching method, attracts participants’ interest in short-term interventions mainly because it is novel and exciting, similar to game-based learning. Yet over time, this sense of novelty diminishes, leading students to become less engaged and even develop negative emotions (such as feeling bored) [35]. In addition, the meta-analysis findings by Li et al [36] found that gamification interventions that were short had a larger mean effect size than those that were long. This suggests that the benefits of short-term gamified interventions are difficult to maintain over time, which explains why the knowledge gained from gamified teaching tends to fade quickly. Furthermore, the delayed effect in the dimension of physical and mental resilience is noteworthy. Students in gamified teaching experienced psychological stress under simulated disaster scenarios, but the development of psychological resilience requires a process of reflection and integration [37]. Kiliç Bayageldi and Kaloğlu Binici [37] pointed out that improvement in psychological qualities in disaster nursing often lags behind the growth of knowledge and skills. In addition, systematic reviews of disaster education also found that improvement in psychological preparedness requires longer intervention and follow-up periods [38].

### The Impact of Gamified Teaching Programs on GSES and Self-Directed Learning Ability

The GSES can effectively measure the confidence of nursing students in responding to disasters. Research has shown that individuals with higher self-efficacy tend to have more positive attitudes, show a stronger willingness to overcome difficulties, and adapt better to new environments [39]. Although the systematic review by Andretta et al [40] reported that gamified teaching can enhance nursing students’ self-efficacy, especially in simulation-based and team-based activities, this study did not find that gamification teaching significantly enhanced GSES scores of students. This contradictory result may be related to the complexity of the disaster nursing environment. In simulated disaster scenarios, students may become more aware of their limitations in knowledge, skills, and psychological preparation within a short period, thus temporarily inhibiting their overall self-confidence [41]. In addition, the choice of evaluation tools may also affect the results. The GSES aims to measure individuals’ general self-confidence across different situations, but it is difficult to accurately capture specific self-confidence in disaster situations [42]. Furthermore, the objectivity of responses obtained through self-reporting faces challenges, as participants may tend to exhibit bias, exaggeration, or even falsification during reporting [35].

Meanwhile, this study revealed clear advantages of gamification teaching in promoting higher-order team collaboration skills. The gamification teaching group consistently outperformed the traditional teaching group in the “learning collaboration” dimension. One possible explanation is that completing the entire learning process in groups within a gamified classroom helps meet students’ need for a sense of connectedness [43]. In addition, gamification may stimulate competition between teams, thereby enhancing participants’ sense of belonging to the team and improving their sense of community affiliation [44]. For example, in the information-gathering phase, team members were required to divide tasks to retrieve relevant materials, and gamification tasks may drive information-processing behavior [12].

### Follow-Up Effects of Gamified Teaching

Compared with some studies that concluded gamified teaching leads to comprehensive improvements in knowledge and skills [45], this study reveals the complexity and context dependence of its effects. Although significant short-term improvements in knowledge and skills were observed in this study (postexperiment scores: GL group>CL group), the 1-week follow-up suggests that consolidation of knowledge retention may require longer-term, repeated interventions or integration with other teaching methods (such as regular drills). This echoes the call for establishing long-term evaluation mechanisms, and future research should pay more attention to the psychological mechanisms in gamification design to meet students’ psychological needs, thereby promoting meaningful and lasting outcomes [46]. In addition, this study incorporated gamification elements into traditional offline classrooms, which may have fostered strong social bonds among participants. Subsequent research could extend this to real-world activity settings [47].

### Comparison to Prior Work

High-quality disaster nursing education can significantly improve response outcomes in mass casualty incidents. The ICN framework emphasizes that standardized education improves professional practice, implicitly supporting innovative teaching that bridges theory and practice [18,19]. Gamified teaching, which introduces game elements such as points, competition, and collaboration into learning activities, has been proposed as a promising strategy [9,10]. Previous research has evidenced the effectiveness of digital game-based interventions in disaster education, demonstrating improvements in knowledge, triage speed, and clinical reasoning [12,14]. However, most existing studies rely on digital gaming platforms or serious games, which require substantial technical resources, limiting their scalability in resource-constrained classroom settings [16,17].

In this study, we used a low-technology, highly interactive classroom gamification approach as the primary method to improve disaster nursing education under conventional teaching conditions. Unlike digital game-based interventions, our approach integrates simple game elements—points, group competitions, and role-play—into existing offline classroom activities. This design provides learners with opportunities to actively engage in simulated disaster scenarios and receive immediate feedback, which may deepen their understanding of disaster response procedures. This mechanism may explain the significant immediate improvement in knowledge and practical skills observed in the GL group.

While some studies argue that short-term gamification impact may be driven by novelty and fade quickly [35], this study does not fully support such a conclusion. Although knowledge and skills showed diminished between-group differences at 1-week follow-up, the physical and mental quality dimension demonstrated a significant delayed effect that emerged only at follow-up, and collaborative learning ability showed sustained improvement that strengthened over time. Therefore, gamified teaching may trigger different learning mechanisms—immediate engagement for knowledge and skills and reflective consolidation for psychological resilience and teamwork—at different temporal stages.

Moreover, while some systematic reviews have reported that gamified teaching can enhance self-efficacy [40], our study did not find a significant effect on general self-efficacy. This contradictory result may be explained by the complexity of disaster scenarios: students in simulated high-stress environments may become more aware of their limitations, temporarily inhibiting general self-confidence [41]. Considering the team-based nature of disaster response, this suggests that low-tech classroom gamification could be a valuable and scalable tool in disaster nursing education, particularly for institutions with limited access to digital simulation resources.

### Practical Significance and Teaching Recommendations

Based on the above findings, we propose the following teaching recommendations: First, gamification can serve as a beneficial supplement to traditional disaster nursing instruction, especially for segments requiring rapid enhancement of knowledge and skills and stimulation of learning motivation. Existing systematic reviews have indicated that gamification teaching can effectively enhance the emergency response and decision-making abilities of nursing students [16]. We recommend arranging gamified activities in the latter part of the course to avoid gaps in foundational knowledge. Second, to maintain long-term effects, gamified review activities should be designed with spaced repetition. Research in cognitive psychology has shown that the spaced repetition strategy can significantly enhance long-term memory retention [48]. For example, an online challenge or group competition can be held 1 week after class to consolidate learning outcomes. Third, cultivating psychological resilience requires giving students time for reflection and internalization. Reflective practice has been proven to enhance students’ emotional resilience and ability to cope with complex situations in nursing education [49]. Teachers can organize sharing sessions or have students write reflective journals after games to help transform stressful experiences into mental toughness. Fourth, strengthen teamwork elements, since cooperation skills are the most notable long-term benefits of gamified teaching. Mechanisms such as cross-group peer evaluations and intergroup challenges can be implemented [43]. Fifth, regarding self-efficacy and self-management skills, relying solely on short-term gamified teaching may be insufficient; it should be combined with mentor feedback, accumulation of successful experiences, and self-directed learning tasks as part of a multifaceted strategy [36].

### Limitations

This study has several limitations. First, it adopted a quasi-experimental design, with each intervention group containing only one class and without individual randomization, which may lead to selection bias. Therefore, the results should be considered exploratory or feasibility evidence. Second, the study was conducted only at one undergraduate institution in Sichuan Province, with a relatively homogeneous sample source, which may limit the generalizability to other regions, institutional levels, and educational cultures. Third, the study mainly assessed short-term and follow-up outcomes, which are insufficient to support conclusions about long-term effects or sustained learning. Future research needs to verify these by extending the follow-up period. Finally, all outcome measures in this study were based on self-reports and did not include objective performance evaluations. Future research could incorporate objective indicators such as behavioral observations or skills assessments to enhance the robustness of the evidence.

### Conclusions

Unlike most existing studies that rely on digital gaming platforms, this study demonstrates that a low-technology, highly interactive classroom gamification approach is a feasible and effective alternative. Research shows that compared with traditional lecture-based teaching, classroom-embedded gamified teaching can more effectively enhance undergraduate nursing students’ disaster nursing competence and self-directed learning ability. In particular, knowledge and skills show marked short-term improvement, physical and mental qualities exhibit a delayed effect, and information literacy and cooperative learning skills improve significantly. However, both teaching models have a limited effect on improving students’ self-management ability and general self-efficacy. This theory-based gamified teaching approach has a low entry threshold and strong interactivity, making it feasible and valuable for promotion under regular teaching conditions. It is suitable for disaster nursing courses and can also serve as a reference for high-pressure health professional education, such as emergency nursing and public health emergency response. Given the limitations of this study, including nonrandom cluster assignment, single-center design, short intervention duration, and lack of objective evaluation, future research should undertake multicenter, randomized controlled trials with long-term follow-up, incorporating disaster-specific psychological indicators to further refine this teaching model.

## Supplementary material

10.2196/85947 Multimedia Appendix 1 Specific teaching objectives and steps.

## Abbreviations

CL: conventional learning
GL: gamified learning
GSES: General Self-Efficacy Scale
ICN: International Council of Nurses
MD: mean differences
TREND: Transparent Reporting of Evaluations with Nonrandomized Designs
UNISDR: United Nations Office for Disaster Risk Reduction
