# Supplementary material for: The Impact of Gamified Teaching on Undergraduate Nursing Students’ Disaster Nursing Competence, Self-Efficacy, and Self-Directed Learning Ability: Quasi-Experimental Study
Source: JMIR Serious Games. 2026 Jun 10;14:e85947. doi: 10.2196/85947 (PMC13252707; doi:10.2196/85947)
Supplement: Multimedia Appendix 1 [file games-v14-e85947-s001.docx]

Objectives of the Gamified Teaching Plan

| Topic | On-Site Rescue Management for Earthquake Disasters | Duration | 180 min | Participants | 66 |
| --- | --- | --- | --- | --- | --- |
| Learning Objectives | 1. Knowledge Objectives:  ① Master the START triage criteria, including the rapid identification of abnormal vital signs (breathing, circulation, and level of consciousness), priority treatment sequences, and triage requirements for special populations (children, pregnant women, and the elderly);  ② Be familiar with disaster response procedures, with a focus on mastering common criteria for on-site casualty classification (such as distinguishing between minor, severe, and critical injuries, as well as fatalities);  ③ Understand the core principles of on-site disaster medical care, including safety assessment, dynamic resource allocation, and the collaborative operational mechanisms of emergency response systems.  2. Skill Objectives:  ① Be able to independently verify the completeness and validity of first aid kit supplies to ensure they meet disaster relief standards;  ② Be able to perform basic first aid procedures, such as hemostatic bandaging and cardiopulmonary resuscitation (CPR), in accordance with established protocols and pass simulation assessments;  ③ Be able to list more than 10 key medical and health resources (e.g., stretchers) in disaster scenarios and explain their uses;  ④ Be able to complete START triage for at least 10 simulated casualties within 15 minutes, with an accuracy rate of over 90%;  ⑤ Be able to complete a simulated group exercise in a real-world disaster scenario through teamwork and write a reflection report of at least 200 words on the rescue process;  ⑥ Ensure the effective operation of rescue procedures during simulated drills (e.g., completing the separation of minor and major casualties within 15 minutes, with a resource mobilization response time of ≤3 minutes).  3. Attitudinal Objectives: ① Possess strong teamwork and interpersonal communication skills;  ② Demonstrate a sense of professional ethics and the ability to provide psychological support;  ③ Exhibit a work ethic characterized by integrity, dedication, scientific rigor, and meticulousness, along with a strong awareness of laws and regulations, nursing ethics, and humanistic care. | | | | |
| Challenges | Time allocation decisions in multitasking scenarios; operational stability under high-pressure conditions; emotional and ethical conflicts. | | | | |
| Implementation Preparation | 1. Materials: Multimedia classroom, emergency care training lab, timer, SuperStarLearn app, manikins, list of first aid supplies, whiteboard, etc.  2. Instructor Preparation: Familiarize yourself with the students’ learning needs and the entire teaching process in advance.  3. Student Preparation: Complete the pre-class preparation and actively participate in classroom activities. The 8 student groups will each take on one stage of the teaching task in pairs, with groups assigned the same task competing against each other. Each group designates 2 members to act as simulated casualties during relevant drills. Two additional students from the whole class are assigned as scorekeepers, rotating across groups. | | | | |
| Background | The earthquake scenario is based on data from the Extreme Events Game provided by LabX. | | | | |

Note: SuperStarLearn APP is an online interactive-teaching and sharing platform with higher popularity in China, which provides comprehensive technical support for remote and short-distance interaction between teachers and students.

The Implementation Process of Gamified Instruction

| Phase 1: Disaster Preparedness Boot Camp (60 min) | | | | |
| --- | --- | --- | --- | --- |
| Instructional Steps – 1. Grouping and Contextual Introduction (5 min) | | | | |
| Teaching Procedure | 1. Distribute “Sector Cards” (containing the START triage method and questions on identifying high-risk individuals) and “Resource Cards” (list of first aid kit supplies) to each group; 2. Project the earthquake scenario background and explain the game rules; 3. Earn points through quick-response quizzes and task challenges, and award prizes at the end. | | | |
| Teacher Responsibilities | 1. Divide the 66 students into 8 groups of 8–9 students each (with 2 simulated casualties per group); 2. Distribute props and explain their uses; 3. Operate the electronic timer (30 seconds per question); 4. Update the leaderboard in real time. | | | |
| Student Tasks | 1. Assign roles within each group (answerer, supply manager, recorder); 2. Submit answers after group discussion; 3. Incorrect answers may be used to spend points for hints. | | | |
| Venue Setup | Display group lists on the multimedia classroom screen; use the SuperStarLearn app uniformly: this integrates buzzer, timing, scoring, and score-tallying functions to reduce manual operations. | | | |
| Instructional Steps – 2: Smart Challenge START Triage Method and Identification of High-Risk Individuals (30 min) | | | | |
| Teaching Procedure | 1. Final Round (15 minutes)  ① Questions regarding the patient’s vital signs are displayed on the projector. Participating teams face away from the screen; when it is their turn to answer, the respondent faces the screen.  ② Respondents must provide their answers verbally within 30 seconds.  2. Collaborative Error Correction (10 minutes)  ① Teams that have not finished their questions may continue to complete the remaining ones, while other teams analyze the incorrect answers.  3. Scoring and Awards (5 minutes)  ① First, compare the number of remaining members; the team with fewer members wins. In the event of a tie, compare total time spent first (the team with the shortest time wins), followed by accuracy rate. | | Game Rules: Correct answer: The contestant leaves the line and earns 10 points; Incorrect answer: The contestant moves to the back of the line to wait for the next round, and other teams may buzz in (correct interrupt to answer earn 5 points; incorrect answers result in a 5-point deduction). Each team must complete 8 questions sequentially. For each question, the designated responder must answer within 30 seconds. The total time for all 8 questions is capped at 4 minutes; unanswered questions receive no points. | |
| Teacher Responsibilities | 1. Start the timer; 2. The instructor provides feedback and clarifies key points; 3. Record the time each group took to answer the questions, their accuracy rate, and the pass rate for equipment preparation; 4. Present the “High-Efficiency Triage Team” award (accuracy rate ≥ 90%). | | | |
| Student Tasks | 1. Quick discussion within the group; 2. Answer the questions under the teacher’s guidance. | | | |
| Venue Setup | The projector displays the questions, and the scoreboard displays the points. | | | |
| Instructional Steps – 3. First Aid Kit Check Challenge (15 min) | | | | |
| Teaching Procedure | 1. Supply Screening  ① Select 10 essential supplies from the 24 Resource Cards (including medications, medical devices, consumables, and item weights);  ② Introduce 10% expired medications, redundant supplies (e.g., 20 packs of cotton swabs), and mismatched medical devices (e.g., surgical scalpels).  2. Thorough Verification  ① Inspect each selected item: verify expiration dates, quantities, and intended uses, and mark any traps;  ② Dynamic Interference: The instructor randomly introduces “emergency situations” (e.g., “A medication is suspected of being contaminated and requires re-verification”).  ③ The team leader reports the intended use of the supplies and the verification results to the instructor. | | Game Rules: Earn 10 points for each correctly identified trap; lose 5 points for each misidentified normal item; earn 2 points per item for a complete description of its use. If a team does not finish within the time limit, 10 points will be deducted for every minute it is late. | |
| Teacher Responsibilities | 1. Manage patient tags (minor/moderate/severe injuries); 2. Record triage times and error rates; 3. Lead the error correction session. | | | |
| Student Tasks | 1. Sort and verify supplies, then fill out the record sheet; 2. Submit reports to earn bonus points. | | | |
| Venue Setup | Instructor: Projector, countdown screen;  Teams: Each team is equipped with a whiteboard, Resource Cards, verification flowchart, and record sheet;  Trap indicators: Expired medications are labeled with red tags; redundant supplies are labeled with yellow tags;  Learning Pass App: Automates countdown, quick response, and score tracking;  Alarm sound effects: Create a tense atmosphere. | | |  |
| 10-Minute Progress Report | | | |  |
| Phase 2: Hands-on Training at a Disaster Site (40 min) | | | |  |
| Instructional Steps – 4: Dynamic Triage Challenge (15 min) | | | |  |
| Teaching Procedure | 1. Ten simulated casualties are brought into the venue; on-site assessment and triage must be completed within the time limit;  2. Sound effects simulating secondary disasters are triggered to evaluate the students’ on-site response;  3. Cross-check the labels assigned by other teams; the team with the lowest error rate receives bonus points;  4. “Triage Expert” medals are awarded.  (15 minutes) | Triage Rules: A flowchart of the START triage method (breathing, circulation, consciousness) is displayed on a screen. The patient manikins are labeled with typical injury categories (e.g., “Difficulty breathing—red,” “Fracture—yellow,” “Abrasions—green”). The triage area is divided into three zones: “Mild,” “Moderate,” and “Severe.” Students are required to place the patients in the appropriate zones based on their labels. | |  |
| Teacher Responsibilities | 1. Manage patient tags (minor/moderate/severe injuries); 2. Record triage times and error rates; 3. Lead the error correction session. | | |  |
| Student Tasks | 1. Quickly assess the casualty’s vital signs and apply a label; 2. The team verifies the results; 3. Submit the triage results. | | |  |
| Venue Setup | The simulation site is equipped with casualty manikins, triage labels, and a timer. | | |  |
|  | Instructional Steps – 5: On-Site First Aid (15 min) | | |  |
| Teaching Procedure | 1. First Aid Procedures: Provide appropriate first aid to 10 injured individuals within the allotted time; the assessment will focus on first aid skills such as hemostasis, CPR, patient transport, and infection control. | Game Rules: A 15-point penalty will be imposed for time-outs or incorrect actions, but some points can be recovered by completing bonus tasks (such as correctly answering questions on first aid, disease prevention, and related topics). | |  |
|  | 2. Environmental Interruptions: Randomly trigger “supply shortages” (e.g., insufficient bandages requiring substitutes); play an “aftershock alert” during the final 2 minutes of the countdown to force participants to speed up their procedures. |  |  |  |
| Teacher Responsibilities | 1. Set environmental interference parameters  2. Monitor operational procedures;  3. Introduce “Team Rescue Cards”: After failing, players can use a card to request support from teammates. | | |  |
| Student Tasks | 1. Perform emergency procedures such as bandaging to stop bleeding and CPR under disruptive conditions;  2. Reallocate resources after a failed attempt. | | |  |
| Venue Setup | 1. Distracting environment simulation area;  2. CPR manikin;  3. First aid kit (including penalty cards). | | |  |
| 10-Minute Progress Report | | | |  |
| Phase 3: Review and Reflection (40 min) | | | |  |
| Teaching Steps – 6: Empathy Communication Workshop (Role-Playing + Application of Empathy Phrases) (30 min) | | | |  |
| Teaching Procedure | 1. Provide four typical conflict scenarios (e.g., family members breaking down emotionally, injured patients refusing treatment, withholding of information, competition for resources, and managing public opinion), with one scenario randomly selected for each group;  2. Introduce “hidden requirements”: Key information is implied in the dialogue of injured patients or family members (e.g., “Should we treat children or adolescents first?”), and students must proactively identify and address these requirements. | Game Rules: The 8 groups are paired into 4 teams. In each pair, one group acts as victims and the other as rescue workers. Each team performs for 5 minutes. The remaining 6 groups (3 pairs) serve as voting observers. Observers will score the performances based on three criteria: “verbal empathy, nonverbal communication (such as body language), and problem-solving.” | |  |
| Teacher Responsibilities | 1. Play a 1-minute video on empathetic communication (e.g., healthcare workers comforting family members) before the role-play; 2. Facilitate the voting session. | | |  |
| Student Tasks | 1. Act as an injured person to express needs; 2. Healthcare workers respond with empathy; 3. Audience members record effective phrases. | | |  |
| Venue Setup | 1. Simulation training room; 2. Hospital beds and props for applying makeup to simulate injuries; 3. Audience seating and voting devices. | | |  |
| 10-Minute Progress Report | | | |  |
| Phase 4: Prevention and Community Action (40 min) | | | |  |
| Teaching Steps – 7: Earthquake Safety Education (30 min) | | | |  |
| Teaching Procedure | 1. Develop educational programs tailored to specific groups (e.g., the elderly, children, people with disabilities, pet owners, etc.);  2. Have residents ask questions based on a pre-set list (e.g., “How do you stay safe during an earthquake in a high-rise building?”). | | |  |
| Teacher Responsibilities | 1. Before the session, students can watch videos demonstrating self-rescue and mutual aid skills for disaster response to gain a basic understanding of community disaster relief; 2. Pose “critical questions” (e.g., “Are these measures really effective?”) to test students’ ability to adapt. | | |  |
| Student Tasks | 1. Explain key points to the “residents”; 3. Optimize the plan based on feedback. | | |  |
| Venue Setup | 1. Community activity simulation area; 2. Educational materials (posters, models); 3. Resident role cards and feedback forms. | | |  |
| 10-Minute Progress Report | | | |  |

Note: Students must complete the assignment within the specified time frame to advance to the next stage.
